# Education Influences Creativity in Dyslexic and Non-Dyslexic Children and Teenagers

**DOI:** 10.1371/journal.pone.0150421

**Published:** 2016-03-07

**Authors:** Zoï Kapoula, Sarah Ruiz, Lisa Spector, Marion Mocorovi, Chrystal Gaertner, Catherine Quilici, Marine Vernet

**Affiliations:** 1 IRIS team Physiopathology of Binocular Motor Control & Vision, FR3636 Neuroscience, CNRS, University Paris Descartes, 45 rue des Saints-Pères, 75006, Paris, France; 2 Ecole de Psychologues Praticiens, 23 rue du Montparnasse, 75006, Paris, France; 3 Collège et Lycée Saint Sulpice, 68 rue d’Assas, 75006, Paris, France; Kyoto University, JAPAN

## Abstract

**Background and Study Hypothesis:**

Are dyslexic children and teenagers more creative than non-dyslexic children and teenagers? Whether creativity is higher in dyslexia, and whether this could be related to neurological development specific to the dyslexic disorder, or to compensatory strategies acquired later in life, remains unclear. Here, we suggest an additional role of differential educational approaches taken in each school that could either enhance or suppress an already higher baseline creativity of dyslexic children and teenagers.

**Results:**

Creativity in dyslexic and non-dyslexic children and teenagers from different schools in France and in Belgium, as well as in students from different universities, was evaluated with the Torrance Test of Creative Thinking (TTCT). Children and teenagers with dyslexia and/or with other similar dysfunctions showed higher creativity scores than non-dyslexic participants. Moreover, the educational approach could further enhance the creative scores in dyslexia, which could be as high as those measured in students from art universities.

**Conclusions:**

We conclude that dyslexic children and teenagers can be highly creative. Yet, expression of creativity can be modulated by educational approach, indicating a probable advantage for personal follow-up compared to normalizing education strategies.

## Introduction

Creativity is difficult to define. Nevertheless, it is generally agreed that creativity is “the ability to produce work that is both original (new, unusual, novel, unexpected) and valuable (useful, good, adaptive, appropriate)” [1, 2]. Paul Torrance is known for developing the Torrance Test of Creative Thinking (TTCT) in 1966 [3]. This American psychologist defined creativity as: “a process of becoming sensitive to problems, deficiencies, gaps in knowledge, missing elements, disharmonies, and so on; identifying the difficulty; searching for solutions, making guesses, or formulating hypotheses about the deficiencies: testing and retesting these hypotheses and possibly modifying and retesting them; and finally communicating the results”. The norms for his test have been adjusted four times (1974, 1984, 1990, and 1998) and translated into more than 35 languages. There are two forms (A and B) of the TTCT-Verbal and two forms (A and B) of the TTCT-Figural tasks. For each task, the stimulus is an image to which people respond by writing (TTCT-Verbal) or drawing (TTCT-Figural). The TTCT measures four factors: *Fluency* shows the ability to produce many figural images (number of relevant ideas). *Flexibility* shows the ability to produce different ideas (number of ideas’ categories). O*riginality* shows the ability to produce uncommon responses (number of statistically non-frequent ideas). Finally, e*laboration* shows the ability to develop and elaborate an idea (number of added details, ideas).

Recently, the neural substrate of creative thinking has been investigated, and an important role of the prefrontal cortex was revealed. Indeed, patients with fronto-temporal degeneration, compared to healthy controls and patients with a non-demented form of Parkinson’s disease, showed impaired creativity scores, which were correlated with impairments in other cognitive tests assessing frontal functions [4]. Other studies revealed that creative thinking could be influenced by other cognitive functions such as mood [5] or motivation [6]. Notably, gender does not seem to influence creativity [7].

Are people with dyslexia particularly creative? What could be the underlying source of such enhanced creativity? Dyslexia is defined as a developmental disorder and hence associated with impairment. Reading ability in dyslexic individuals is significantly lower than what could be expected from the intelligence quotient. However, the existence of superior skills in dyslexic individuals has been hypothesized. Examples of particularly creative individuals who happened to be dyslexic are abundant (see e.g., [8]). However, it remains unclear in which domains dyslexic individuals might be superior and what could be the basis of such enhanced skills.

The scientific literature shows mixed results with regards to enhanced visuo-spatial skills and creativity in dyslexia. Early studies reported that higher creativity was observed in adults, but not in children [9]. Concerning higher visuo-spatial abilities in dyslexic children and teenagers, conflicting findings from the literature were related to the type of ability being tested [10, 11]. Dyslexic children or teenagers were better at evaluating impossible figures [11] or detecting visuo-spatial tasks in a virtual reality environment [10], suggesting that holistic or real-life visuo-spatial information processing are among the specific skills enhanced in dyslexia. In addition, young children recently diagnosed as dyslexic were shown to be better at generating navigation signs and symbols when asked to produce communication designs [12]. The observation that more dyslexic college students are found in artistic than in non-artistic fields adds further to the evidence favoring a potential link between dyslexia and creativity [13].

Several hypotheses have been proposed to explain enhanced skills in dyslexia. The first type of argument speculates that there must be an evolutionary advantage, which maintained such a high prevalence of dyslexia in the current human population. A potentially more efficient parvocellular system in response to a weaker magnocellular system [14], or a developmental delay of the dominant hemisphere, most likely disinhibiting the non-dominant parietal lobe [15], have been suggested. The second type of argument speculates that higher creativity arises later in life as a compensation for early failure and with the development of unconventional coping strategies (see [13]). In any case, the important role of education has been underlined, and it has been suggested that children with learning disorders should be encouraged to develop hidden talents instead of being subjected to overemphasizing their deficits [15].

In the present study, we reexamined the potentially higher creativity in dyslexic children and teenagers taking into account the type of education (specificity vs. normalization). We hypothesized that schools, which adapt their education approach to the need of dyslexic children and teenagers would promote creativity as measured by the TTCT. The present study was conducted with children and teenagers from three different schools in France and Belgium with different educative approaches. As a comparison, we also measured creativity in students from three universities, which promote different types of creativity (decorative art, industrial creation and design, and engineering).

## Materials and Methods

### Participants

The first part of the study was conducted with three groups of students (young adults) from three universities in Paris, including a university devoted to decorative art: *Ecole Nationale Supérieure des Arts Décoratifs* (ENSAD), a university devoted to industrial creation and design: *Ecole Nationale Supérieure de Créations Industrielles* (ENSCI), and an engineering university: *Ecole Nationale Supérieure des Techniques Avancées* (ENSTA-ParisTech). The second part of the study was conducted with children and teenagers from three schools in France and Belgium, including a school in Paris, France (the school will be simply referred here as PARIS), in which we recruited children and teenagers with dyslexia and with other dysfunctions, a school in Bruxelles, Belgium, (the school will be referred as BRUXELLES), in which we recruited dyslexic and non-dyslexic children and teenagers, and a school in Oise, France (the school will be referred as OISE), in which we recruited dyslexic children with or without comorbidity. Demographic data can be found in Table 1. The schools were selected because they offered special programs for dyslexic children and teenagers and because the teachers were open to participate in our research study. Sample size of each group was limited by the number of children, teenagers, and students, who agreed to participate in the study. Previous studies were able to show differences between groups in creativity using the TTCT test with approximately 10 participants in each group (as low as 7 in some cases) [16–18]; all our statistical analyses, except one, which will be indicated, were performed with a larger sample size. Written informed consent was obtained from the students and from both the children/teenagers and their parents. The study was approved by the local ethical committee C*onseil d’évaluation éthique pour les recherches en santé* (CERES) and was conducted in adherence with the Declaration of Helsinki.

**Table 1:** Demographic data.

University / School	Mean Age	STD Age	Min Age	Max Age	Males	Females
**ENSAD**	**26.3**	**2.9**	**20**	**27**	**6**	**3**
**ENSTA**	**22.6**	**1.4**	**21**	**25**	**6**	**2**
**ENSCI**	**21.3**	**1.8**	**18**	**24**	**3**	**5**
**PARIS**	**12.5**	**0.8**	**11**	**14**	**47**	**19**
*dyslexic*	*12*.*5*	*0*.*9*	*11*	*14*	*38*	*16*
*other dysfunctions*	*12*.*3*	*0*.*8*	*11*	*14*	*9*	*3*
**Bruxelles**	**13.3**	**0.9**	**12**	**15**	**20**	**21**
*dyslexic*	*13*.*7*	*1*.*0*	*12*	*15*	*8*	*7*
*Non-dyslexic*	*13*.*3*	*0*.*9*	*12*	*15*	*12*	*14*
**OISE**	**10.3**	**1.3**	**8**	**12**	**9**	**1**
*dyslexic without comorbidity*	*10*.*5*	*1*.*3*	*9*	*12*	*4*	*0*
*dyslexic with comorbidity*	*10*.*2*	*1*.*3*	*8*	*12*	*5*	*1*

The young-adult students (in ENSAD, ENSTA, ENSCI) were never diagnosed as dyslexic. However, several students (3/9) from the ENSAD expressed having had school difficulties when they were young such as: mixing up letters, reading difficulties, attention deficits, pronounciation difficulties. In the PARIS school, children and teenagers had either dyslexia (n = 54) or other dysfunctions (n = 12, including 4 with single and 8 with multiple difficulties: dyspraxia (2), dysphasia (2), attention deficit (3), dysgraphia (1), written language difficulties (5), oral language difficulties (2), cognitive inhibition (1)). In the BRUXELLES school, children and teenagers were either dyslexic (n = 15) or non-dyslexic (n = 26). In the OISE school, all recruited children had dyslexia, some without (n = 4), and some with comorbid dysfunctions (n = 6; 3 with dysphasia, 2 with attention problems and 1 with dyscalculia).

Children and teenagers were classified as dyslexic independently from the research team by specialized schools, medical centers, or children’s hospital services according to criteria commonly used in France and Belgium. The dyslexia classification was based on an extensive examination including neurological, psychological, and phonological capabilities, performed less than a year before being included in the present study. In particular, reading abilities were evaluated using the L2MA battery [19], a standard test commonly used for evaluating oral and written language, memory and attention of French-speaking children in France and Belgium. This battery includes an evaluation of reading speed, text comprehension, phonological fluency, visual naming, passive lexical stock, irregular words and pseudo-words reading, and spelling. The ability to use phonetic skills to decode words is specifically assessed using the pseudo-words reading test. Inclusion criteria were: (1) scores in the L2MA test were two standard deviations below the normal score, (2) a normal intelligence quotient, and (3) no neurological symptoms or ophthalmologic pathology. The authors knew which children and teenagers were classified as dyslexic or non-dyslexic, but did not have access to individual clinical test results. We would like to emphasize that although intelligence and neurological evaluations were normal for all participants, we cannot entirely rule out the probability that they could be confounding factors in the present study.

### Tests of creativity

**Fig 1:**
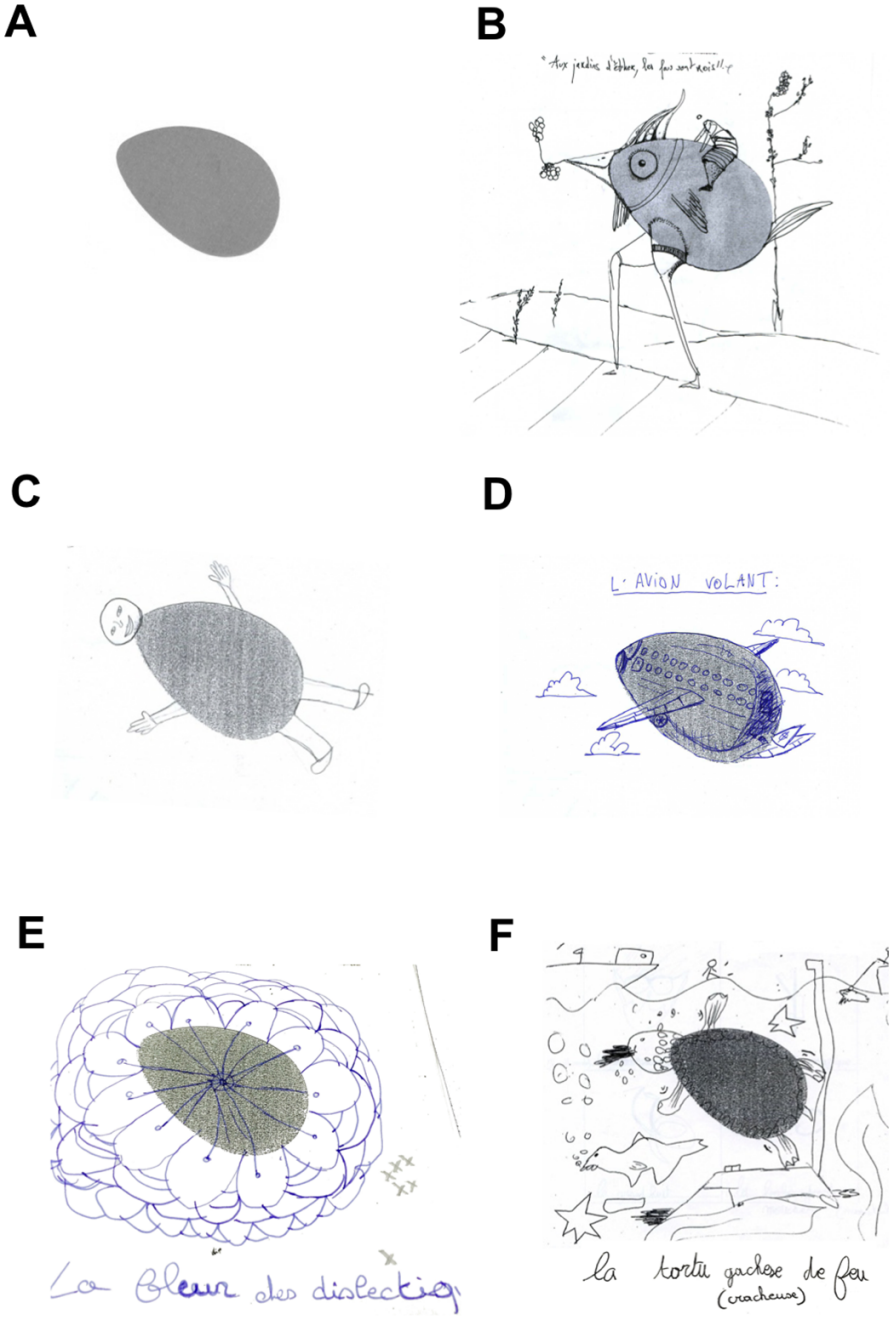
Illustration of the TTCT completion for representative participants. (A) Original form to be completed. (B) Completion of a student from ENSAD (fluency: 55; flexibility: 45; originality: 60; elaboration: 75); title “*Aux jardins d’éther*, *les fous sont rois*”. (C) Completion of a non-dyslexic 14 years old teenager from BRUXELLES (fluency: 35; flexibility: 35; originality: 35; elaboration: 40); title “*Garçon*”. (D) Completion of a dyslexic 13 years old teenager from BRUXELLES (fluency: 65; flexibility: 65; originality: 65; elaboration: 75); title “*L’avion volant*”. (E) Completion of a dyslexic 12 years old child from PARIS (fluency: 40; flexibility: 30; originality: 40; elaboration: 50); title “*La fleur des dislectiq*”. (F) Completion of a dyslexic 12 years old child from OISE (fluency: 60; flexibility: 65; originality: 70; elaboration: 55); title “*La tortu gachese [cracheuse] de feu*”.

The tests were run by school personnel, who were trained by the investigators., Participants of each university/school were simultaneously administered the TTCT–Figural Form test in a quiet room. The paper-pencil test was handed out to each participant, together with clear instructions. The test was started after everyone had understood the instructions. The TTCT–Figural Form test, is an age-normed (up to 18 years old) test composed of three tasks, each lasting 10 min; all require producing unusual drawings starting from standard shapes (e.g., a pair of straight lines or an oval, see Fig 1). The results were analyzed by three authors, who are students in psychology and trained in the analysis of the Torrance test; the authors knew which school the children attended, but were blinded to their classification as being dyslexic or non-dyslexic. The scores assessed four different cognitive components of creativity: fluency (the quantity of relevant productions), flexibility (the number of different categories of productions), originality (the degree to which productions are uncommon), and elaboration (the degree of enrichment of productions). Each component yielded a raw score, which was then converted into a standard score with a calibrated chart. The data are available upon request to the corresponding author.

### Data analysis

One-way ANOVAs were separately performed for each creativity score (fluency, flexibility, originality, elaboration, and finally the total score) for testing the effect of the following factor: university (students from the 3 universities), dyslexia (dyslexic vs. non dyslexic children and teenagers from the BRUXELLES school), school (dyslexic children and teenagers from the 3 schools), age (dyslexic children and teenagers from the 3 schools, or non-dyslexic children and teenagers from the BRUXELLES school), type of dysfunction (children and teenagers with dyslexia vs. with other dysfunctions from the PARIS school) and comorbidity problems (dyslexic children and teenagers with or without comorbidity problems from the OISE school). Finally, ANOVAs were used to compare the most creative dyslexic children and teenagers (i.e., from the BRUXELLES school, see results) with the most creative students (i.e., from the ENSAD university, see results). Post-hoc analyses were performed with the Least Significant Difference (LSD) test. Tests were two-tailed. Statistical significance was set at p < 0.05. Confidence intervals (CI) and Cohen’s d effect size are indicated.

## Results

One part of the study was conducted with students from three universities in Paris, attending courses on decorative art (ENSAD), industrial creation and design (ENSCI), and engineering (ENSTA-ParisTech). The second part of the study was conducted with children and teenagers from three schools in France and Belgium, hereafter named PARIS (pure dyslexia and dyslexia with other dysfunctions), BRUXELLES (with and without dyslexia) and OISE (dyslexia with or without comorbidity problems). Creativity of all participants was tested with the TTCT–Figural Form test. The quotation assessed four different cognitive components of creativity: fluency, flexibility, originality, and elaboration, and finally, a combined total score.

### Effect of the university on the creativity of students

**Fig 2:**
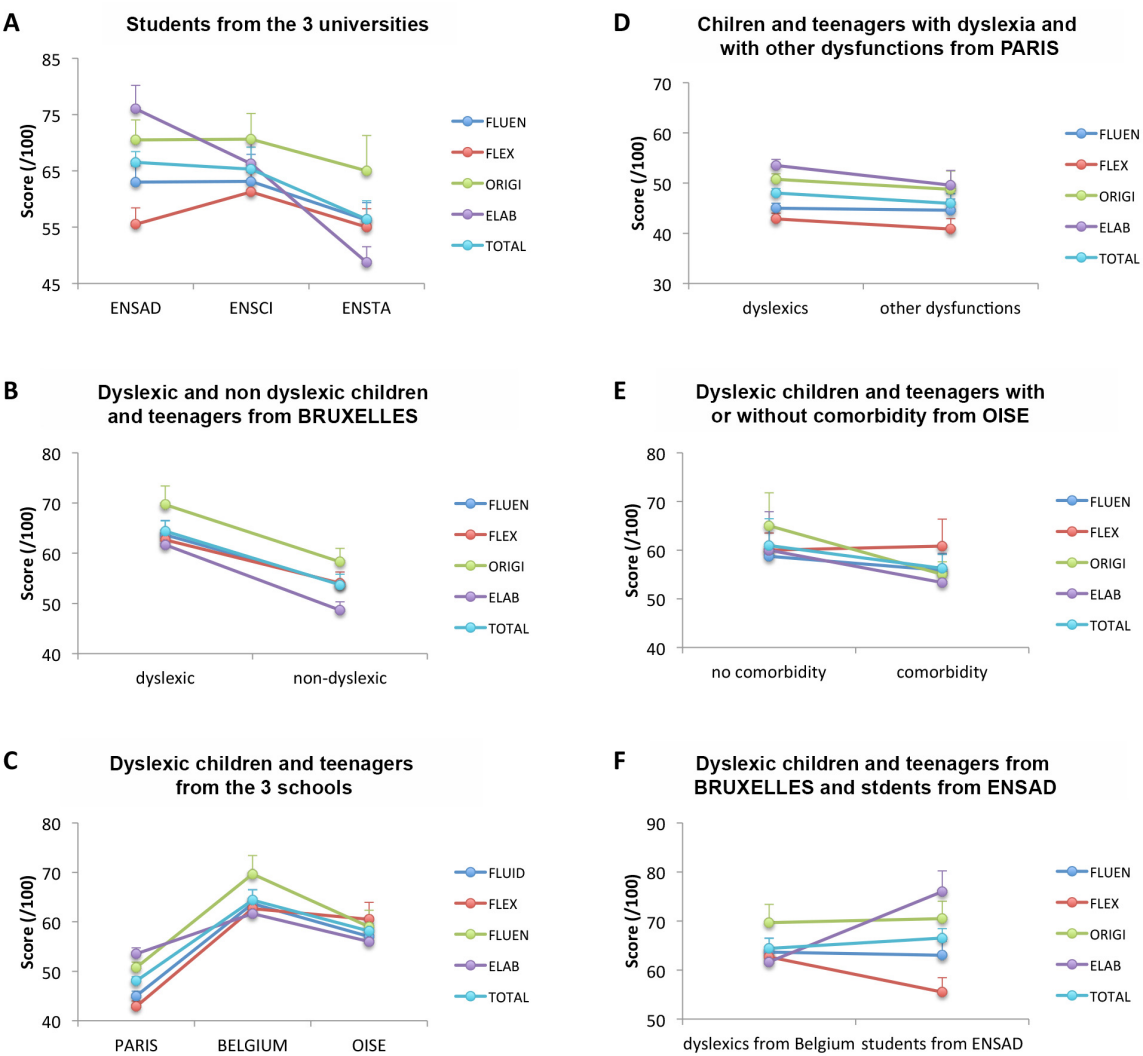
TTCT results. TTCT results for each components of creativity: fluency (FLUEN), flexibility (FLEX), originality (ORIGI) and elaboration (ELAB) as well as total score (TOTAL) when testing for university effect among students (A), when testing for dyslexia effect among children and teenagers (B), when testing for school effect among dyslexic children and teenagers (C), when comparing children and teenagers with dyslexia and with other dysfunctions (D), when comparing dyslexic children and teenagers with and without comorbidity (E) and when comparing dyslexic children and teenagers from BRUXELLES with students from ENSAD (F).

The scores of the students from the three different universities (Fig 2A, Table 2) revealed lower elaboration and total scores for ENSTA students than for ENSAD and/or ENSCI students. Recall that the TTCT is calibrated up to 18 years, and the students tested in the present study were beyond this age. Yet, the observed differences are of interest, and in line with Kim’s suggestion [20] that such a test can be useful for research as it has the capacity to differentiate between groups. Thus, students attending a university valorizing artistic or industrial creation displayed higher creativity than students enrolled in engineering.

**Table 2:** Effect of the university on the creativity of students.

	F(2,22)	p	CI	d
**ANOVA**				
Fluency	0.96	>0.250		
Flexibility	1.15	>0.250		
Originality	0.42	>0.250		
Elaboration	11.58	**<0.001** ***		
Total	3.02	**0.0696** '		
**ENSAD vs. ENSCI**				
Elaboration		0.1334	[-3.57 24.41]	0.39
Total		>0.250	[-8.19 10.12]	0.06
**ENSAD vs. ENSTA**				
Elaboration		**<0.001** ***	[15.99 39.84]	1.28
Total		**0.0206** *	[1.74 18.00]	0.63
**ENSCI vs. ENSTA**				
Elaboration		**0.0058** **	[5.95 29.05]	0.84
Total		0.102	[-2.01 19.82]	0.44

': p <0.07 (marginally significant);

*: p<0.05;

**: p<0.01;

*** p<0.001;

CI: 95% confidence interval; d: Cohen’s d effect size (interpretation: d = 0.2: small; d = 0.5: medium; d = 0.8: large effect size).

### Dyslexia and creativity

Comparing the scores of dyslexic and non-dyslexic children and teenagers from the BRUXELLES school (Fig 2B, Table 3) revealed a significant main effect of dyslexia on all parameters measured. Note that both groups were very comparable in age (see Table 1). Thus, the dyslexic children and teenagers were evaluated as more creative than the non-dyslexic participants.

**Table 3:** Effect of dyslexia on creativity (BRUXELLES).

	F(1,39)	p	CI	d
**ANOVA**				
Fluency	6.46	0.015 *	[2.00 17.63]	0.42
Flexibility	7.00	0.012 *	[2.03 15.22]	0.46
Originality	6.28	0.017 *	[2.20 20.59]	0.40
Elaboration	21.21	<0.001 ***	[7.30 18.73]	0.74
Total	11.35	0.0017 **	[4.28 17.12]	0.58

See Table 2 for symbols’ legend and interpretation.

### Effect of the school on the creativity of dyslexic children and teenagers

Comparing the scores of dyslexic children and teenagers from the 3 schools (Fig 2C, Table 4) revealed that all scores were significantly higher in BRUXELLES than in PARIS and that all scores except the elaboration score were significantly higher in OISE than in PARIS. Finally, none of the scores were significantly different in BRUXELLES when comparing to OISE. Note that test scores were normed according to the age and were therefore not biased by age. Thus, the educational approach had an impact on creativity in dyslexic children and teenagers.

**Table 4:** Effect of school on creativity.

	F(2,76)	p	CI	d
**ANOVA**				
Fluency	34.69	<0.001 ***		
Flexibility	42.53	<0.001 ***		
Originality	22.41	<0.001 ***		
Elaboration	4.11	0.020 *		
Total	35.88	<0.001 ***		
**BRUXELLES vs. PARIS**				
Fluency		<0.001 ***	[13.91 23.43]	1.02
Flexibility		<0.001 ***	[15.06 24.53]	1.27
Originality		<0.001 ***	[13.24 24.62]	0.84
Elaboration		0.0026 **	[2.96 13.34]	0.46
Total		<0.001 ***	[12.44 20.29]	1.14
**OISE vs. PARIS**				
Fluency		<0.001 ***	[6.93 17.07]	0.78
Flexibility		<0.001 ***	[11.60 23.65]	0.92
Originality		<0.001 ***	[2.42 14.10]	0.44
Elaboration		>0.250	[-4.36 9.32]	0.11
Total		<0.001 ***	[5.42 14.76]	0.67
**BRUXELLES vs. OISE**				
Fluency		0.12	[-1.84 15.17]	0.34
Flexibility		>0.250	[-5.34 9.67]	0.12
Originality		0.057	[-0.35 21.68]	0.43
Elaboration		0.240	[-4.01 15.43]	0.24
Total		0.074	[-0.67 13.22]	0.38

See Table 2 for symbols’ legend and interpretation.

### Influence of age in creativity

**Fig 3:**
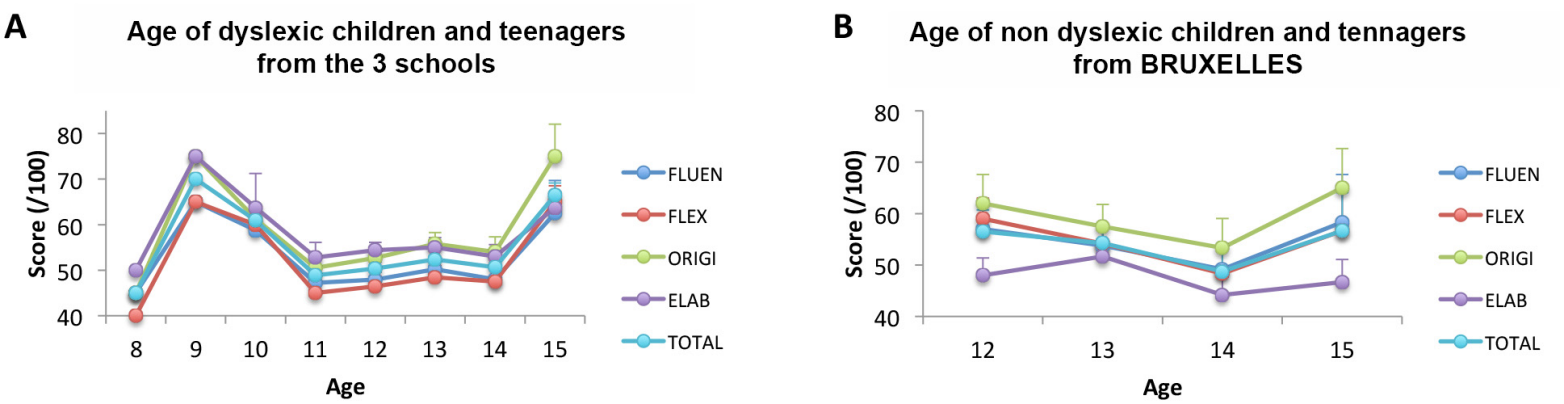
TTCT results per age category. TTCT results per age category for dyslexic children from the 3 schools (A) and for non dyslexic children from BRUXELLES (B).

Indeed, there was no age effect on any of the scores in non-dyslexic children and teenagers from BRUXELLES (Fig 3B, Table 5). On the contrary, a significant age effect was found for dyslexic children and teenagers from the three schools. In brief, there are critical time periods in dyslexic children and teenagers during which higher scores are obtained: 10 and 15 years old (Fig 3A, Table 6). Most probably, such age effects did not affect the fact that they were higher scores for dyslexic children and teenagers in the BRUXELLES school than in the PARIS school (see previous section): the average age of both groups was between 12 and 14 years old, i.e., the age ranges with the lowest creativity score in dyslexia.

**Table 5:** Effect of age on the creativity of non-dyslexic children and teenagers.

	F(3,22)	p
**ANOVA**		
Fluency	0.5	>0.250
Flexibility	0.87	>0.250
Originality	0.6	>0.250
Elaboration	1.13	>0.250
Total	0.6	>0.250

**Table 6:** Effect of age on the creativity of dyslexic children and teenagers.

	F(7,71)	p	CI	d
**ANOVA**				
Fluency	1.7	0.12		
Flexibility	2.33	0.034 *		
Originality	2.85	0.011 *		
Elaboration	1.64	0.13		
Total	3.09	0.0066 **		
**10 vs. 11**				
Flexibility		0.033 *	[1.47 28.52]	0.79
Originality		0.0597	[-0.52 21.91]	0.69
Total		0.011 *	[3.33 20.76]	0.91
**10 vs. 12**				
Flexibility		0.026 *	[1.77 25.31]	0.71
Originality		0.1374	[-2.91 20.00]	0.50
Total		0.008 **	[3.00 18.14]	0.78
**15 vs. 11**				
Flexibility		0.0067 **	[6.77 33.23]	1.11
Originality		0.003 **	[10.22 38.67]	1.05
Total		<0.001 ***	[0.34 25.88]	1.48
**15 vs. 12**				
Flexibility		0.0031 **	[6.87 30.21]	1.03
Originality		0.001 **	[9.88 34.70]	0.90
Total		<0.001 ***	[8.72 23.56]	1.33
**15 vs. 13**				
Flexibility		0.022 *	[2.61 30.47]	0.82
Originality		0.010 *	[4.92 33.54]	0.71
Total		0.021 *	[2.32 15.96]	0.86
**15 vs. 14**				
Flexibility		0.014 *	[4.28 30.72]	0.91
Originality		0.0095 **	[6.16 35.84]	0.78
Total		0.0078 **	[5.02 26.73]	0.97

See Table 2 for symbols’ legend and interpretation. NB. For the post-hoc analysis, the 8 and 9 years old group containing only 2 children were excluded and only pairs of ages showing statistically significant differences are reported.

### Comparison of creativity in children with dyslexia and/or other associated dysfunctions

In the PARIS school, children and teenagers with dyslexia and those with other dysfunctions had similar creativity scores (Fig 2D, Table 7). In the OISE school, dyslexic children and teenagers with and without comorbid dysfunctions also had similar creativity scores (Fig 2E, Table 8). However, the sample size of this last analysis might have been too small to allow detecting differences between children with or without comorbid dysfunctions. If confirmed in larger groups, those results would indicate that the specific type of developmental dysfunction does not impact creativity. Such findings would reinforce the probable importance of the educational approach taken by the school.

**Table 7:** Effect of the type of impairment (dyslexia vs. other dysfunction) on creativity (PARIS).

	F(1,64)	p	CI	d
**ANOVA**				
Fluency	0.03	>0.250	[-4.25 5.08]	0.03
Flexibility	0.61	>0.250	[-3.17 7.25]	0.13
Originality	0.48	>0.250	[-3.77 7.75]	0.10
Elaboration	1.83	0.18	[-1.87 9.74]	0.21
Total	1.03	>0.250	[-2.02 6.21]	0.16

See Table 2 for symbols’ legend and interpretation.

**Table 8:** Effect of the comorbidity (with vs. without) on creativity in dyslexic children and teenagers (OISE).

	F(1,8)	p	CI	d
**ANOVA**				
Fluency	0.28	>0.250	[-9.86 15.70]	0.17
Flexibility	0.01	>0.250	[-18.05 16.39]	0.04
Originality	2.56	0.15	[-4.41 24.41]	0.50
Elaboration	0.47	>0.250	[-15.87 29.20]	0.22
Total	0.67	>0.250	[-8.51 17.89]	0.26

See Table 2 for symbols’ legend and interpretation.

### Comparison of creativity in Belgian dyslexic children and teenagers with creativity in art students

Finally, when comparing the most creative group of students (i.e., from ENSAD) and the most creative group of dyslexic children and teenagers (i.e., from BRUXELLES) (Fig 2F, Table 9), the only significant differences were the following: higher flexibility for dyslexic children and teenagers than art students and higher elaboration for art students than dyslexic children and teenagers; there were no significant differences in fluidity, originality, and total scores. It is worth noting that several students (3/9) from the ENSAD school expressed having had school difficulties such as: mixing up letters, reading difficulties, attention deficits, and pronounciation difficulties. Thus, dyslexic children and teenagers in some schools might display the same level of creativity than students in art. Conversely, it is possible that some of the art students experienced dysfunctions similar to dyslexia when they were young.

**Table 9:** Comparison of creativity in Belgian dyslexic children and teenagers with creativity in art students.

	F(1,22)	p	CI	d
**ANOVA**				
Fluency	0.02	>0.250	[-10.99 6.97]	0.10
Flexibility	4.87	0.038 *	[-14.87–0.46]	0.45
Originality	0.02	>0.250	[-10.97 12.75]	0.03
Elaboration	10.38	0.0039 **	[5.34 24.66]	0.65
Total	0.36	>0.250	[-4.57 8.33]	0.13

See Table 2 for symbols’ legend and interpretation.

## Discussion

Our study showed that art students obtain higher scores in the TTCT than engineering students. Therefore, in addition to its capacity in differentiating groups, the TTCT reflects some type of artistic creativity expressed by art-trained students. Even if they were not diagnosed as dyslexic, one third of the art students expressed having had learning difficulties at school. This observation is in line with prior studies showing a high prevalence of dyslexia among art students [13].

Of interest, similar scores of creativity were obtained by art-trained students and dyslexic children and teenagers from the BRUXELLES school, suggesting that dyslexic children and teenagers can be as creative as the student population selected for their creativity and trained to further develop it. Furthermore, our analyses reveal a tendency that, on the one hand, children and teenagers with dyslexia and/or with other similar dysfunctions obtain higher creativity scores than non-dyslexic children and teenagers and that, on the other hand, the educational approach a school chooses can further enhance creativity in dyslexic children and teenagers. Further studies with higher sample sizes would be required to confirm the role of educational systems in enhancing the creativity of dyslexic children and teenagers.

The first result of our study is the finding that dyslexic children and teenagers can possess higher creativity. We therefore moderate the statement that higher creativity is only expressed in dyslexic adults and hence results from compensatory mechanisms initiated in response to the specific difficulties associated with dyslexia [9]. Indeed, dyslexic children around the age of 10 years old were found to be particularly creative in our study, presumably before compensatory mechanisms could be fully developed. We thus suggest that higher creativity in dyslexia partially relies on a neurophysiological basis (e.g., developmentally different balance/interactions between right/left hemispheres or between magnocellular and parvocellular systems [14, 15, 21, 22]), possibly mediating higher holistic visuo-spatial processing skills [10, 11]).

The second result of our study is that the educative environment plays an important role in the development of creativity in dyslexic individuals, a finding that is in line with previous literature [15]. What could be the main reasons explaining differences between schools? General cultural or educational policy most likely differ between France and Belgium, but note that creativity remained higher in the dyslexic when compared with the non-dyslexic population in BRUXELLES. In addition, creativity was similar in the BRUXELLES (Belgium) and OISE (France) schools, and larger in these two schools than in the PARIS school (France) in dyslexic children and teenagers. Our interpretation of the present study’s results is rather that the educational approach targeted to the dyslexic population has an impact on creativity. In terms of educational program, the three establishments are driven by programs that take into account the specificities of the dyslexic population, providing additional help for reading performance. However, some differences in the different establishments may account for differences.

For instance, the PARIS school mostly aims at normalizing reading and academic performance. In order to acquire standard levels of performance in language, dyslexic children and teenagers attend special classes with small numbers of pupils (less than 18), where they benefit from additional hours of training in language skills. Furthermore, orthophonists assess progress outside normal school hours and regular meetings between teachers, parents, othophonists, and school psychologists are scheduled to evaluate the progress.

In the BRUXELLES school, educational emphasis is placed on the specific needs of the individual rather than on a normalization process. The goal is to not discriminate between pupils with and without learning difficulties and to provide a general education approach that takes into account individual differences. For instance, pupils learn how to set their own objectives, and are helped to discover their limits and abilities, and to mobilize their resources to overcome difficulties. Most of the teachers in this school received further training (e.g., in coaching, relaxation, and other techniques).

The OISE school is driven by a similar educational approach. Professionals at this school pointed out the importance of individually pacing and following up each child and teenager in order to improve their reading skills. In order to reconcile dyslexic children and teenagers with school, they attend regular classes where they are not separated and stigmatized. In addition, they attend special classes where they receive additional help from orthophonists, ergotherapists, and psychologists. The teachers in this school included persons educated to teach the general population as well as persons specialized in teaching pupils with dyslexia. Importantly, both the teachers running regular classes and specialized teachers were interacting within the same school, aiming for a common educational goal to improve the performance of dyslexic children and teenagers based on a personalized follow-up.

We believe that all these aspects are of importance and could condition the expression of creativity as measured by the TTCT in different populations. From the present study, we cannot estimate which of these factors could be crucial for potentiating higher creativity in dyslexic children and teenagers, and we cannot rule out other confounding factors, such as class size. Nevertheless, our interpretation in terms of education is in line with the theory of Sternberg [23] suggesting that creativity is also a “decision” and that society can play a role by teaching creative thinking especially to children who profit less from conventional educational approaches.

In conclusion, our study demonstrates that education, which is specifically adapted to the needs of subjects with dyslexia, can enhance creativity in dyslexic children and teenagers. We hope that this study will stimulate further multidisciplinary studies in order to better assess the differences in educational approaches and their impact on expressions of creativity.
